# Crushing Responses of Expanded Polypropylene Foam

**DOI:** 10.3390/polym15092059

**Published:** 2023-04-26

**Authors:** Yueqing Xing, Deqiang Sun, Meiyun Zhang, Guowei Shu

**Affiliations:** College of Bioresources Chemical and Materials Engineering, Shaanxi University of Science & Technology, Xi’an 710021, China; wampire@126.com (D.S.); myzhang@sust.edu.cn (M.Z.); shuguowei@gmail.com (G.S.)

**Keywords:** expanded polypropylene, density, thickness, static compression, cushioning property, optimal cushioning design

## Abstract

This paper aimed to experimentally clarify the crushing mechanism and performance of expanded polypropylene foam (EPP) and analyze the influence of density and thickness on its mechanical behavior and energy absorption properties under static crushing loadings. Hence, a series of compression tests were carried out on EPP foams with different densities and thicknesses. For foam with a density of 60 kg/m^3^, the mean crushing strength, energy absorption (E_a)_, energy absorption efficiency (E_f_), specific energy absorption (SEA), and energy absorption per unit volume (w) increased by 245.3%, 187.2%, 42.3%, 54.3%, and 242.8%, respectively, compared to foam with a density of 20 kg/m^3^. Meanwhile, compared to foam with a thickness of 30 mm, the mean crushing strength, energy absorption (*E*_a_), energy absorption efficiency (*E*_f_), SEA, and energy absorption per unit volume (*w*) for foam with a thickness of 75 mm increased by 53.3%, 25.2%, −10.8%, −4.7%, and −10.6%, respectively. The results show that foam density has a significantly greater influence on static compressive performance than foam thickness. The microstructures of the EPP foam before and after static compression were compared by observing with a scanning electron microscope (SEM), and the failure mechanism was analyzed. Results showed that the load and energy as well as the deformation and instability processes of its cells were transferred layer by layer. The influence of density on the degree of destruction of the internal cells was obvious. Due to its larger mass and larger internal damping, thicker foams were less damaged, and less deformation was produced. Additionally, the EPP foam exhibited a considerable ability to recover after compression.

## 1. Introduction

EPP foam is characterized by being lightweight, with high-heat insulation properties, high specific strength, and excellent mechanical properties. It is commonly used as an energy-absorbing material, and thus has a wide range of applications, especially in the field of cushion packaging [1,2,3,4,5,6,7]. At present, the research focus on EPP foam mainly involves its production and preparation technology, with few studies investigating systematic cushioning properties by the energy method or the deformation process and microstructures observed by SEM. Owing to their lightweight design, EPP foams with low densities are endowed with considerable potential to further increase energy absorption, which has recently drawn increasing attention [8].

Liu [9] experimentally studied the effects of EPP foams with different densities for EPP-filled Al honeycombs under axial and lateral loadings. The authors found that the peak strength, mean crushing strength, and total energy absorption (EA) increased, whereas the specific energy absorption (SEA) remained unchanged with increasing densities of foam fillers in the axial tests. Lee et al. [10] explored the dynamic mechanical properties of EPP foams with three different densities under different strain rates. Cronin et al. [11] found that EPP foam exhibited excellent impact resistance as well as high energy absorption, insulation, and heat resistance. Goods et al. [12] conducted tensile, static compression, and dynamic impact tests for the (EPU). Du et al. [13] found that thicker EPS material resulted in better static buffering performance, while more energy absorption indicated a larger, non-recoverable residual shape variable. David [14] analyzed the composition of plastic foam and pointed out the cushioning mechanism of the foam materials, claiming that when they were forced, the gases in the polymer pores compressed and pores within the cavity allowed large deformations of the material, while stress remained unchanged to absorb external energies. Some other researchers [15,16,17,18] discovered that the mechanical properties and cushioning energy absorption characteristics of the plastic foam were mainly affected by its thickness, density, pore cell structure parameters, environmental temperature, and other factors. Ye Chenxuan et al. [19] studied the cushioning performance of EVA with different densities and thicknesses under dynamic impact compression loading and found that in the case of the same strain values, the stress increased with density and thickness, the thickest sample material had the largest shape variables, and the bearing capacity of the material increased with its thickness and density.

The density, thickness, environmental temperature, humidity, and strain rate of the cushioning material all affect the static cushioning performance of plastic foams and honeycomb paperboard. However, the deformation mechanism and energy absorption characteristics of EPP foam during the static compression process have not been systematically studied. Thus, it is necessary to examine the static cushioning performance of EPP, establish an evaluation system of the static cushioning performance with energy absorption as the core, and analyze the influence of density and thickness on its mechanical behavior and energy absorption characteristics. By analyzing the microstructures of the EPP foams, observed by using a scanning electron microscope (SEM) before and after compression, the damage–deformation process and internal mechanisms were hereby studied. These data will provide theoretical guidance for the practice of cushioning optimization design.

## 2. Materials and Methods

### 2.1. Materials

The EPP foam hereby used as a polymer matrix was acquired from Suzhou Shunsheng Packaging Material Co., Ltd., Suzhou, China, with densities of 20 kg/m^3^, 30 kg/m^3^, 45 kg/m^3^, and 60 kg/m^3^. The length and width of the specimens were 160 mm × 160 mm. The material of each density had five different thicknesses, i.e., 15 mm, 30 mm, 45 mm, 60 mm, and 75 mm, respectively. Specimen labels include a letter and 2 numbers: for example, S represents the uniform code of the test sample, the following two-digit number is the density, and the last two-digit number is the thickness. Thus, specimen S60-30 is an EPP foam with a density of 60 kg/m^3^ and a thickness of 30 mm.

For characterization of pore morphology, the samples were quenched in liquid nitrogen, sprayed with gold, and observed using a JSM6390LV scanning electron microscope produced by Japan JEOL Corporation.

### 2.2. Static Compression Test Equipment

A CMT4303 electronic universal material testing machine with a measuring range of 10 KN and an accuracy grade of 0.5 was used for the test, which was made by Xian Dingnuo Measurement and Control Equipment Co., Ltd., Xian, China. The experimental method was in strict accordance with GB/T 8168-2008 [20], and static compression test specimens were prepared for more than 24 h in an environment of 20 °C and 56% relative humidity. 

During the test, two pieces of steel plates are placed on the upper and lower surfaces of the specimen, respectively. To ensure that the sample is forced by uniform stress, the size of the steel plate should be larger than that of the tested sample. The test should be completed to obtain stress values twice that of the initial peak stress. Quasi-static compression tests on the EPP foams are performed on the height direction at room temperature using the CMT4303 machine. The specimen is loaded at a constant rate of 12 mm/min with a crushing distance of 18 mm to 45 mm [21]. The test automatically stops when overall dimensional deformation reaches 80% of the sample size [22]. The load and displacement data are recorded directly by the automatic data acquisition system. The stress (defined as force over original cross-sectional area) and strain (defined as displacement over original thickness) were calculated so that the peak crushing strength, energy absorption (EA), and specific energy absorption (SEA) could be subsequently calculated based on the stress–strain curves.

### 2.3. Compression Characteristic Criteria

A stress–strain curve is used to analyze the mechanical behavior and energy absorption properties of materials. Several key compression indicators were hereby quantified to allow a comparative study system and evaluate the performance of the EPP foams.

The cushioning coefficient refers to the ratio of the stress to the energy absorption per unit volume of the cushioning material; it is expressed by *C* [23], with a smaller cushioning coefficient indicating greater cushioning efficiency and better static cushioning performance. When the stress increases monotonically with the strain *ε*, such as in EPP foams, the cushioning coefficient *C* can be expressed as:

(1) $$C=\frac{\sigma_{m}}{E_{0}}$$

(2) $$E_{0}=\int_{0}^{\varepsilon }\sigma d\varepsilon$$

where *σ_m_* is the maximum static stress (MPa) and *E*_0_ represents the deformation energy per unit volume of the cushioning material (J).

Miltz et al. [24] proposed the use of energy absorption efficiency *E*_f_ (efficiency) and ideal energy absorption efficiency *I* (ideality) to evaluate the energy absorption characteristics of polymer porous materials. The energy absorption efficiency *E*_f_ at a certain strain *ε*_a_ refers to the ratio of the energy absorbed by the real foam specimen (when compressed to the maximum strain *ε*_a_) to the energy absorbed by an ideal foam specimen with the same size (when fully compressed) and transmits the same maximum stress *σ*_a_, where *σ*_a_ is the compressive stress corresponding to *ε*_a_. This relationship can be described and defined as:

(3) $$E_{f}\left(\varepsilon_{a}\right)=\frac{\int_{0}^{\varepsilon_{a}}\sigma \left(\varepsilon \right)d\varepsilon }{\sigma_{a}}$$

(4) $$e=\int_{0}^{\varepsilon }\sigma_{\left(\varepsilon \right)}d\varepsilon \;\;\;\;0\leq \varepsilon_{a}\leq 1$$

(5) $$E_{f}\left(\varepsilon_{a}\right)=\frac{e}{\sigma_{a}}$$

**Figure 1:**
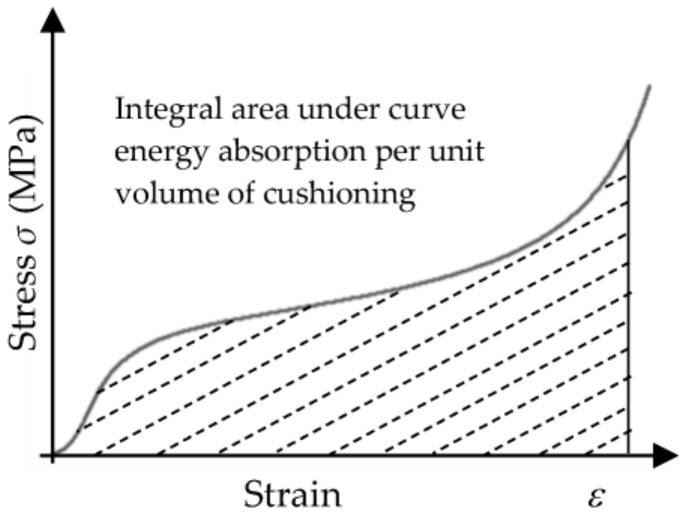
Energy absorption per unit volume from the stress–strain curve.

The unit volume absorption energy *e* is determined by integrating the area below the stress–strain curve. It can be seen that the slope of the curve is constantly changing, and the curve is not monotonous, so the piece-wise method was hereby adopted to obtain the total area of the graph under the curve, as shown in Figure 1.

The cushioning energy absorption capacity of the EPP foams can also be measured in terms of the ideal energy absorption efficiency (*I*), which is defined and described as:

(6) $$I=\frac{\int_{0}^{\varepsilon_{a}}\sigma d\varepsilon }{\sigma_{a}\varepsilon_{a}}$$

The peak crushing strength, *σ*_max_ (corresponding to the peak load *P*_max_ in the force–displacement curve [25] over the cross-sectional area), is a key indicator to assess the load-bearing capacity. It typically represents the initial peak crushing force and corresponds to the collapse of the weakest layer of cells first crushed in compression of the EPP foam [26].

The densification strain, *ε*_D_, is the critical strain when the cell walls are squeezed together, which can be expressed by several methods [27]. It was hereby defined as the maximum value of *ε*_i_, and should satisfy the following condition of maximum efficiency [28]:

(7) $$\frac{dE_{f}\left(\varepsilon_{i}\right)}{d\varepsilon_{i}}\left|\varepsilon_{i}=\varepsilon_{D}\right.\;\;\;0\leq \varepsilon_{i}\leq 1$$

Thus, the mean crushing strength is defined as:

(8) $$\sigma_{m}=\frac{1}{\varepsilon_{D}}\int_{0}^{\varepsilon_{D}}\sigma_{\left(\varepsilon_{i}\right)}d\varepsilon \;\;\;\;\;\;0\leq \varepsilon_{i}\leq 1$$

The total amount of energy absorption through a crushing or bending element is determined by integrating the area below the load–displacement curve, as follows:

(9) $$E_{a}=\int_{0}^{l}Pdl$$

where *E*_a_ is the absorbed energy, *P* is the instantaneous crushing force, and l is the crushing displacement.

Specific energy absorption (*SEA*) is an important factor to compare the energy absorption capacity of different structures. It can be determined by dividing the total energy absorption by the mass of the structure, defined and described as [9]:

(10) $$SEA=\frac{E_{a}}{m}$$

where *E_a_* represents the absorbed energy and *m* is the mass of the specimen.

The area included under the stress–strain curve is the energy absorption per unit volume of the sample, which can be calculated by the following formula:

(11) $$w=\int_{0}^{\varepsilon }\sigma \left(\varepsilon \right)d\varepsilon$$

Therefore, the energy absorption per unit volume corresponding to the densification strain *ε*_D_ is the maximum energy absorption per unit volume.

## 3. Results and Discussion

**Figure 2:**
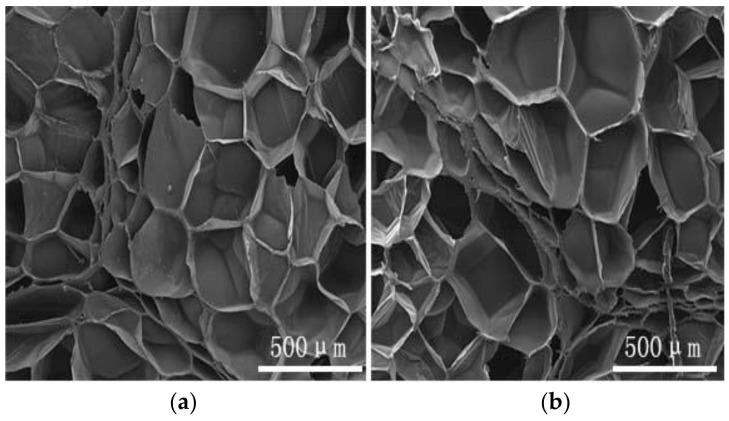
Microstructures (**a**) transverse section; and (**b**) axial section.

We used SEM to observe the microstructures of a transverse and axial section of a specimen of EPP foam with a density of 30 kg/m^3^, magnified 100 times. The EPP foam was hereby demonstrated to be a typical isotropic closed-cell polymer porous material, as shown in Figure 2.

**Figure 3:**
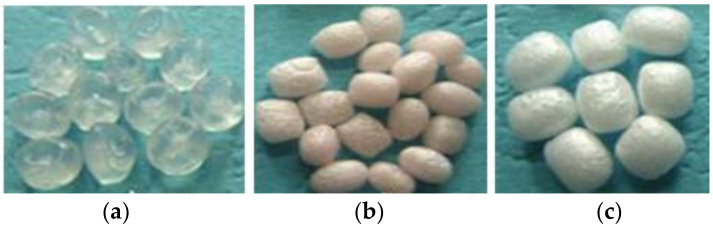
Polypropylene cellular material [30] (**a**) polypropylene resin particle; (**b**) a foaming rate of 15; and (**c**) a foaming rate of 45.

Polypropylene resin particles before undergoing the foaming process are shown in Figure 3a. EPP foam particles with a foaming rate of 15 are shown in Figure 3b, and particles with a foaming rate of 45 are shown in Figure 3c. The tested EPP was foamed by the scCO_2_ autoclaved foaming method. In this process, a high-pressure reaction tube is used with supercritical carbon dioxide (scCO_2_) as the foaming agent. After a certain time of heating pressure, the sudden release of pressure produces the resin foam. The density of the polypropylene resin particles is 900 kg/m^3^, the density of the foam with a foaming rate of 15 is 60 kg/m^3^, and that of the foam with a foaming rate of 45 is 20 kg/m^3^. The void porosity of EPP foam with a foaming rate of 15, 20, 30, and 45 is 93.3%, 95%, 96.7%, and 97.8%, respectively. The void porosity can significantly affect the static compression performance of plastic foams, especially the compression strength [29].

For each of the investigated specimens, tests were carried out at least three times; the average values were considered the final results. The specimens used and the test results are summarized in Table 1.

### 3.1. Stress–Strain Curve and Its Mechanical Model

**Figure 4:**
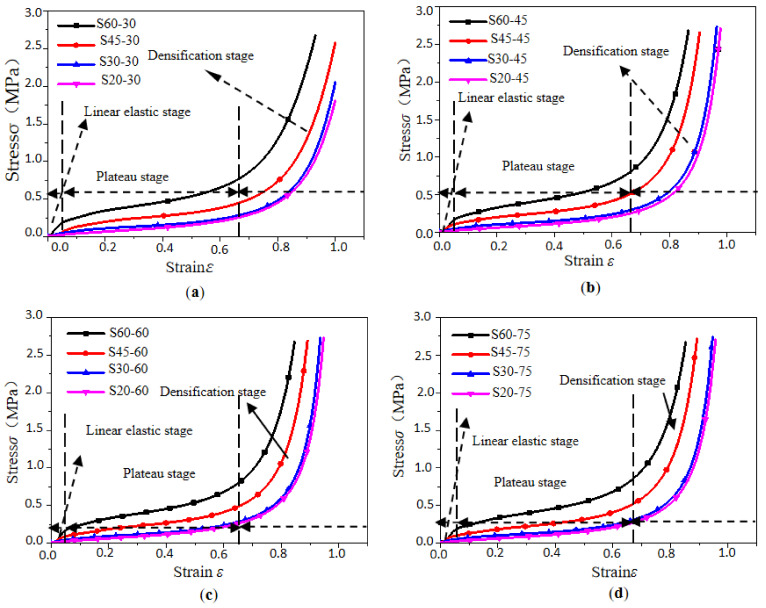
Stress–strain curves of specimens with different densities: (**a**) *t* = 30 mm; (**b**) *t* = 45 mm; (**c**) *t* = 60 mm; and (**d**) *t* = 75 mm.

**Figure 5:**
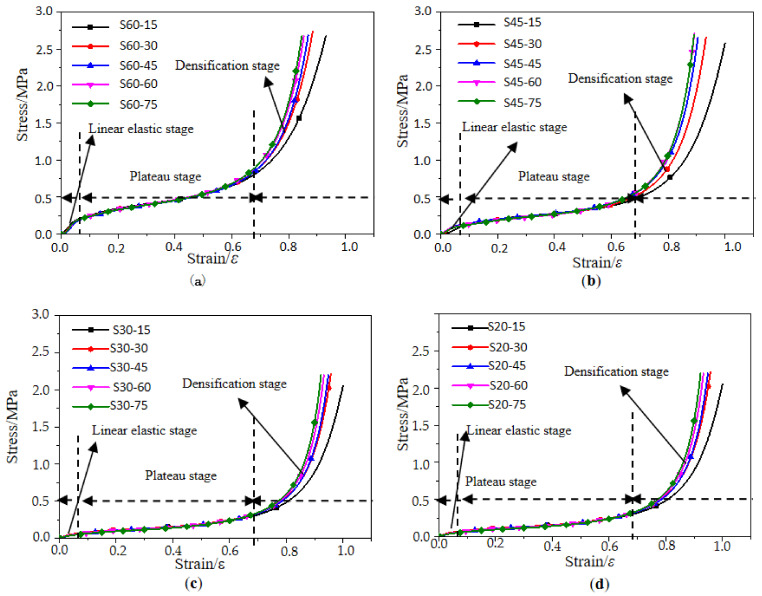
Stress–strain curves of EPP specimens with different thicknesses: (**a**) *ρ* = 60 kg/m^3^; (**b**) *ρ* = 45 kg/m^3^; (**c**) *ρ* = 30 kg/m^3^; and (**d**) *ρ* = 20 kg/m^3^.

The typical stress–strain curves of the EPP foams are drawn in Figure 4 and Figure 5. Similar to the PU foam in other studies [31,32], these curves presented an elastic behavior, with little deformation at the beginning, followed by a plateau region in which the stress was almost constant, and then a densification region where the stress increased rapidly followed by a further increase in strain caused by squeezing the void content inside the foam.

The stress corresponding to the intersection point of the linear fitting line in the elastic region and the linear fitting line in the plateau region is the yield stress, and the corresponding point of the yield stress on the compression curve is the yield point. Herein, the yield point and initial point of densification were determined using the tangential method proposed by Viot P [33].

The results showed that the stress–strain curves of samples of different densities were relatively dispersed, while those of samples with different thicknesses were almost identical. The density of the material had a more significant influence on the static cushioning properties than the thickness. The stress–strain curve was divided into three stages, i.e., the linear elastic stage, the plateau stage, and the densification stage. The stress–strain curves of specimens with different thicknesses had the same or overlapping trends at the linear elastic stage and the plateau stage. The elastic modulus and the initial peak stress of the specimens with the same density were almost the same. In the densification stage, the dispersion trend appeared because of the internal damping. The linear elastic stage was rather short. The plateau stage was longer and was also the main energy-absorbing stage. After this stage, the cells were all pressed together, and the cell wall material was compressed. Therefore, the curve increased sharply, and then the plateau stage transitioned into the densification stage. Though the increment in density was almost the same, the difference between the stress–strain curves of samples with densities of 60 and 45 kg/m^3^ was substantially greater than that between curves with densities of 45 and 30 kg/m^3^. The difference between the stress–strain curves of samples with densities of 20 and 30 kg/m^3^ was minimal, indicating that the greater the density of the sample was, the greater the influence on the stress–strain curve would be. The stress of foam with a density of 60 kg/m^3^ was more than three times that of foam with a density of 20 kg/m^3^, with a larger density indicating a shorter plateau stage and a steeper stress–strain curve.

According to the stress–strain curves, data-fitting analysis was carried out by SPSS software on three kinds of EPP foams: with a density of 20 kg/m^3^ and a thickness of 30 mm, a density of 60 kg/m^3^ and a thickness of 30 mm, and a density of 60 kg/m^3^ and a thickness of 60 mm. The constitutive relation models of stress related to strain were obtained, respectively, as:

(12) $$\sigma_{1}=3.597\varepsilon_{1}^{3}-4.437\varepsilon_{1}^{2}+1.943\varepsilon_{1}+0.003$$

(13) $$\sigma_{2}=4.545\varepsilon_{2}^{3}-4.721\varepsilon_{2}^{2}+1.636\varepsilon_{2}-0.54$$

(14) $$\sigma_{3}=5.694\varepsilon_{3}^{3}-6.469\varepsilon_{3}^{2}+2.560\varepsilon_{3}-0.036$$

The values of the goodness-of-fit coefficient R^2^ of the above three curves are 0.992, 0.980, and 0.989, respectively, indicating a favorable model-fitting effect. According to the above constitutive relation models, the stress–strain curve of EPP foam is a cubic function with a constant term.

**Figure 6:**
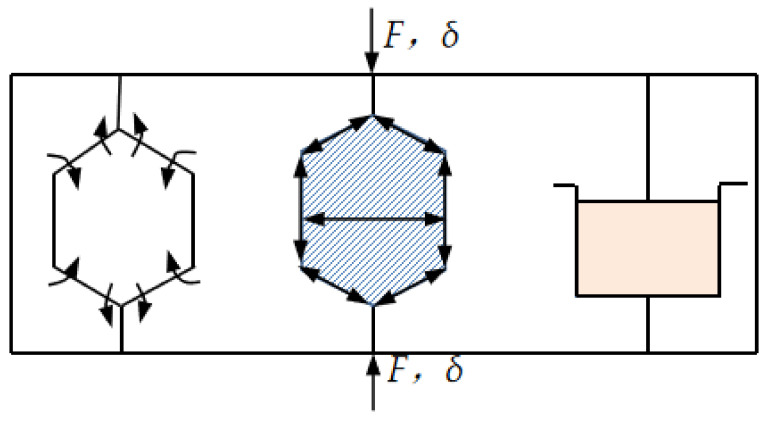
Deformation mechanism map of the EPP closed cell: wall bending + wall edge contraction and membrane extension + enclosed gas pressure.

It can be seen from Figure 6 that when the EPP closed-cell foam was exerted by force F and produced displacement δ, the deformation was mainly composed of three parts, i.e., cell-wall bending, cell-wall edge contraction and membrane extension, and enclosed gas pressure, respectively. The effect of the gas pressure improved the rigidity of the closed-cell foam and led to an increase of Young’s modulus to a certain extent.

**Figure 7:**
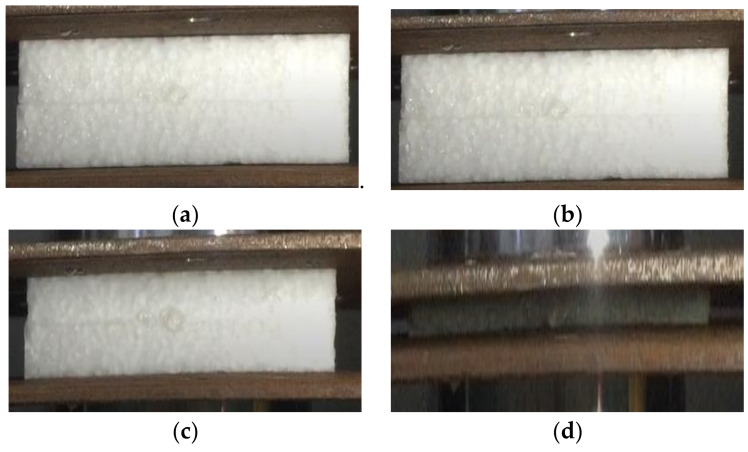
Compression deformation process of EPP foams. (**a**) Before compression (ε = 0); (**b**) linear elastic stage (ε = 1%); (**c**) plateau stage (ε = 5%); and (**d**) densification stage (ε = 7%).

The whole experimental process was recorded using a high-speed camera to observe the deformation instability process, as shown in Figure 7. The specimen was observed before compression, as shown in Figure 7a. At the beginning of the linear elastic stage of deformation, the foam was compressed to a strain of 1%, as shown in Figure 7b. The foam began to deform slightly from the pressure head close to the pressure plate. At the plateau stage, the foam was compressed to a strain of 5%, as shown in Figure 7c. After a long period of stress strengthening in the plateau stage, the foam absorbed most of the energy, deformed greatly, and entered the densification stage, as shown in Figure 7d. At this time, the sample was gradually compacted, and the test was completed.

### 3.2. Microstructures of the EPP Foam Observed by SEM before and after Static Compression

**Figure 8:**
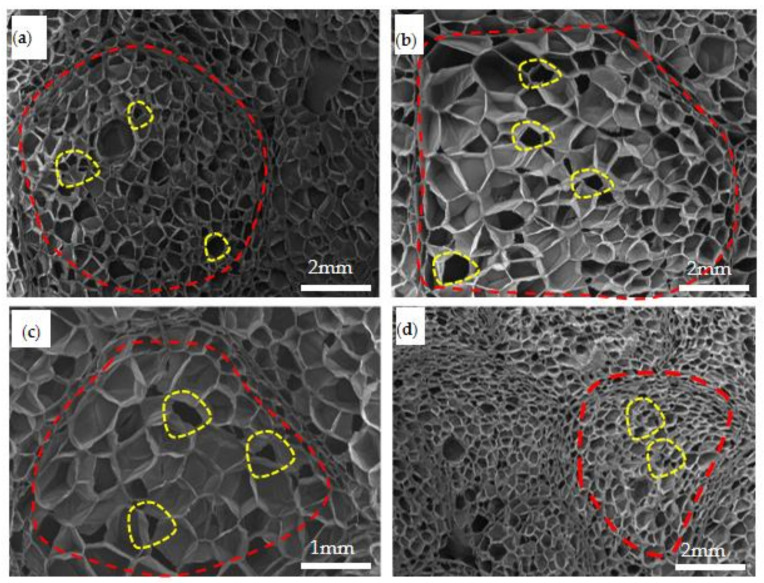
The axial section SEM structure of samples with different densities before compression at (**a**) *ρ* = 45 kg/m^3^; (**b**) *ρ* = 20 kg/m^3^; (**c**) *ρ* = 30 kg/m^3^; and (**d**) *ρ*= 60 kg/m^3^. (The area circled in red line is a foaming particle; the area circle in yellow line is a pore.)

The axial section of microstructures before static compression from specimens with different densities was observed by SEM, as shown in Figure 8. The EPP foam is generally composed of many foaming particles with different sizes and shapes (the area circled in red line is a foaming particle), and each foaming particle has many cells in its internal structure. It can be seen from the size and shape of the foam particles within the area outlined by a red dotted line in Figure 8 that the shape of foam particles was irregular, spherical, hemispherical, polygonal, or elliptic. The average diameter of foaming particles with a density of 20 kg/m^3^, 30 kg/m^3^, 45 kg/m^3^, and 60 kg/m^3^ was about 3~4 mm, 2.5~3 mm, 2~2.5 mm, and 1~1.5 mm, respectively, with higher-density foam consisting of smaller cell sizes and a larger number of cells. A single foaming particle of each density EPP foams had a number of pores(the yellow dotted line in Figure 8), and the pore shape was variable. These expanded beads were then injected into a steam chest mould, where they fused together under steam heat and pressure. The cell gas expanded the beads, which agglomerated and fused together to form the structure of the foam. The density of each moulded part depended on the number of porous beads injected into the mould, and the microstructure of these foams varied with the density.

**Figure 9:**
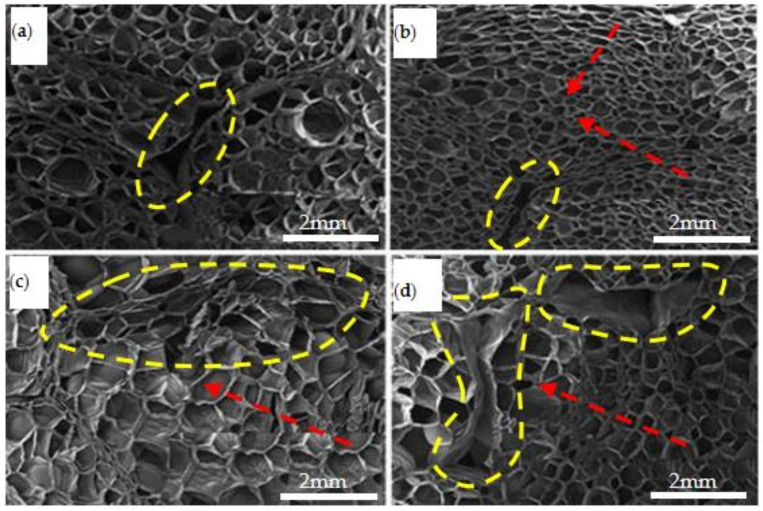
The axial section SEM structure after compression (**a**) *ρ* = 60 kg/m^3^, t = 30 mm; (**b**) *ρ* = 45 kg/m^3^, t = 60 mm; (**c**) *ρ* = 30 kg/m^3^, t = 60 mm; and (**d**) *ρ* = 20 kg/m^3^, t = 60 mm. (The red arrow line was the compression force transfer direction; the yellow dotted lines were somewhat wrinkled and broken walls.)

After static compression, the foams were cut in the axial direction, and their microstructures were observed by SEM and analyzed, as shown in Figure 9. It can be seen that the cells close to the pressure head collapsed first, and those cell walls began to fold. Then, the load was gradually transferred to the cells of the next layer. Thus, when EPP foams are subjected to static compression load, the load and energy is transferred layer by layer, as is the deformation and instability process of its cells.

The compression started from the cells at the edge of the particles and gradually transferred to the center of the cells, as shown in Figure 9b. The direction indicated by the red arrow is the compression force transfer direction. Meanwhile, after static compression, deformation started to decrease and some cells in the center of the foam particles even returned to their original shape when they gradually transitioned to the central cells.

The area outlined by the yellow dotted line in Figure 9b shows where the cell walls were less damaged, and the cells in the center of the foam particles were basically undamaged. As shown in Figure 9c, there were some folds on the cell walls at the junction of the foaming particles, and the cell wall at the edge of the foam particle was destroyed (inside the yellow dotted line). In Figure 9d, the cells of the lowest density foam were damaged the most seriously, with the appearance of many folds and cracks in the cell body junction. Some of the cell walls were squeezed together. Moreover, this kind of deformation could not be recovered after the removal of the load, resulting in permanent deformation. The influence of density on the degree of destruction of internal cells was obvious.

As demonstrated by the yellow dotted line in Figure 9a, some cell walls at the edge of the particles were somewhat wrinkled and broken, and the cell walls in the center of the foaming particles were wrinkled and partially collapsed. As shown in Figure 9b, the cell walls at the edge of particles were exposed to little deformation, while there were almost no wrinkles and collapse on the cells in the center of the particles. Due to their larger mass and larger internal damping, thicker foams were less damaged, and less deformation was produced. Thus, thickness had a certain effect on the internal structures after compression. It was found that after compression, the EPP foam cells could be recovered to a certain extent and could bear additional impact loadings; on the contrary, the PU foam was completely destroyed and was no longer able to absorb energy. Therefore, the EPP foam was considered to have more potential to be used for the energy absorption bumpers [9].

### 3.3. Diagram of Total Energy Absorption and Specific Energy Absorption

**Figure 10:**
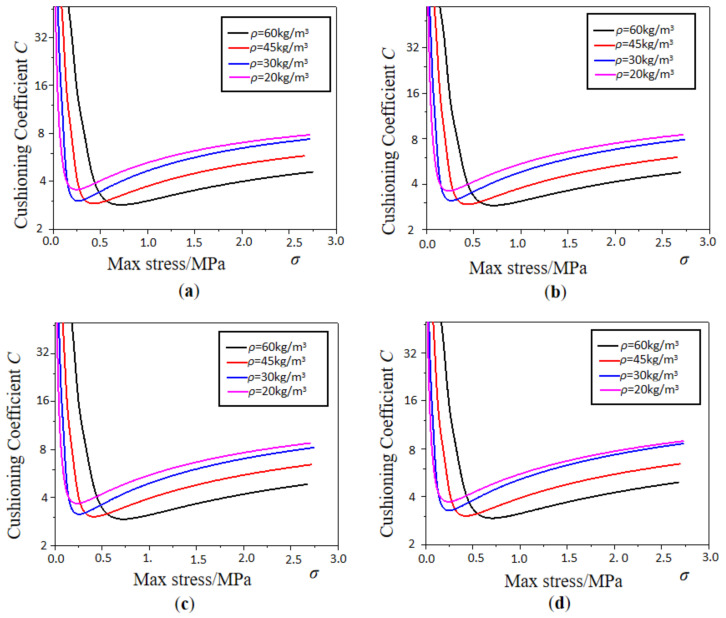
Cushioning coefficient–max stress curves of EPP foams with different densities: (**a**) *t* = 30 mm; (**b**) *t* = 45 mm; (**c**) *t* = 60 mm; and (**d**) *t* = 75 mm.

**Figure 11:**
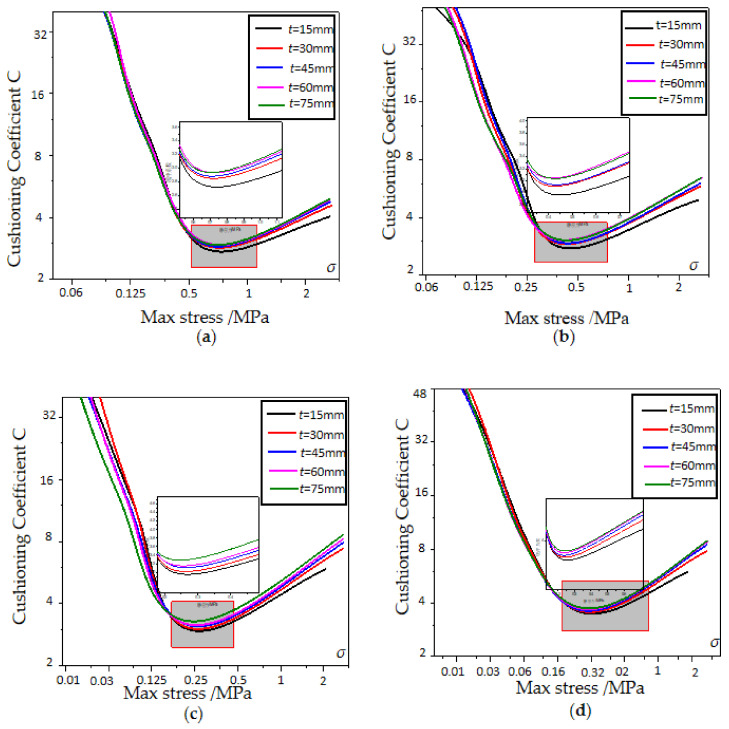
Cushioning coefficient—max stress curves of EPP foams with different thicknesses. (**a**) *ρ* = 60 kg/m^3^; (**b**) *ρ* = 45 kg/m^3^; (**c**) *ρ* = 30 kg/m^3^; (**d**) *ρ* = 20 kg/m^3^.

The cushioning coefficient and maximum stress curves of EPP foam with different densities and thicknesses are shown in Figure 10 and Figure 11, respectively. Under the condition of the same density, the minimum cushioning coefficient decreases with increasing density, and the density of 60 kg/m^3^ has the minimum cushioning coefficient and the best cushioning performance compared with other densities. Under the condition of the same density, the EPP foam with a thickness of 15 mm at the same static stress has the smallest cushioning coefficient. Therefore, the static cushioning performance of the EPP foam with a thickness of 15 mm is better than that of the other four thicknesses specimens. Meanwhile, it can be seen from the curves that the minimum cushioning coefficient difference between foams with different thicknesses is rather small, and thickness has little effect on the cushioning coefficient.

**Figure 12:**
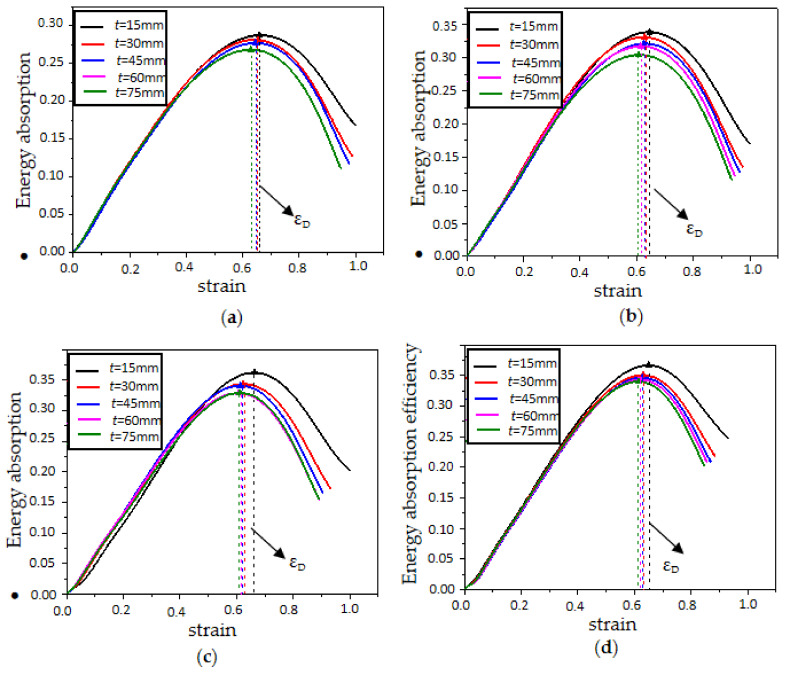
Efficiency–strain curves of samples with different thicknesses: (**a**) *ρ* = 20 kg/m^3^; (**b**) *ρ* = 30 kg/m^3^; (**c**) *ρ* = 45 kg/m^3^; and (**d**) *ρ* = 60 kg/m^3^.

**Figure 13:**
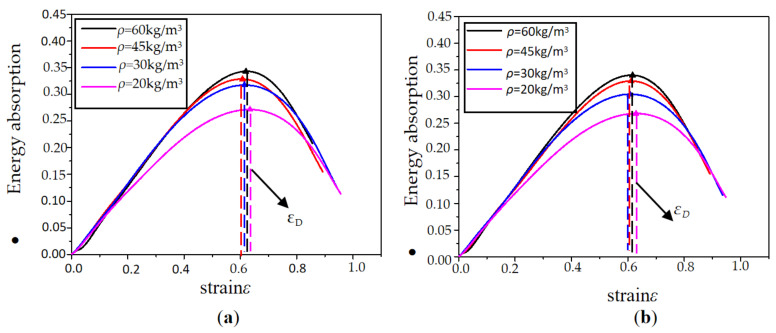
Efficiency–strain curves of samples with different densities: (**a**) *t* = 60 mm; (**b**) *t* = 75 mm.

The energy absorption efficiency–strain curve is depicted in Figure 12 and Figure 13. The density increased from 20 kg/m^3^ to 60 kg/m^3^, and the energy absorption efficiency of the EPP foam materials increased by 42.3% (0.2612–0.3467). The values of energy absorption efficiency corresponding to foam thicknesses of 15 mm, 30 mm, 45 mm, 60 mm, and 75 mm were, in turn, 0.3667, 0.3510, 0.3467, 0.3467, and 0.3400. The EPP foam thickness ranged from 15 mm to 75 mm, and the energy absorption efficiency decreased by 10.8% (0.3667–0.3400).

The maximum point of efficiency curves for foam densities of 45 kg/m^3^ and 60 kg/m^3^ was located above the curves for foam densities of 30 kg/m^3^ and 20 kg/m^3^, and the maximum point of efficiency curves with thicknesses of 15 mm and 30 mm was located above the curves with thicknesses of 60 mm and 75 mm, indicating that the denser and thinner EPP foam provides a higher energy absorption efficiency.

**Figure 14:**
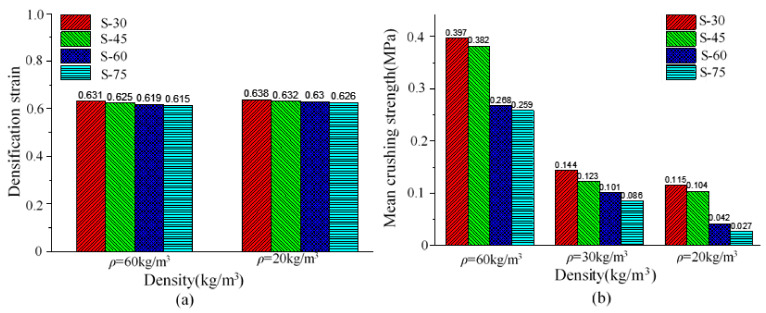
Effect of density and thickness on loading bearing capacity:(**a**) Densification Strain (**b**) Mean crushing stress.

It should also be noted that the densification strain was not very sensitive to foam density or thickness. As shown in Figure 14, the densification strain was about 0.6 to 0.7 for all EPP foams. It can be observed that density had a significant influence on the mean crushing strength of the EPP foams. As shown in Figure 14b, when EPP foam density increased from 20 kg/m^3^ to 60 kg/m^3^, the mean crushing strength increased by 245.2% (0.115 MPa–0.397 MPa). As foam thickness increased from 30 mm to 75 mm, the mean crushing strength increased by 53.3% (0.259 MPa–0.397 MPa). The influence of foam density on energy absorption efficiency and mean crushing strength had also been found in the static axial compression performance of honeycomb panels filled with EPP foams [9].

**Figure 15:**
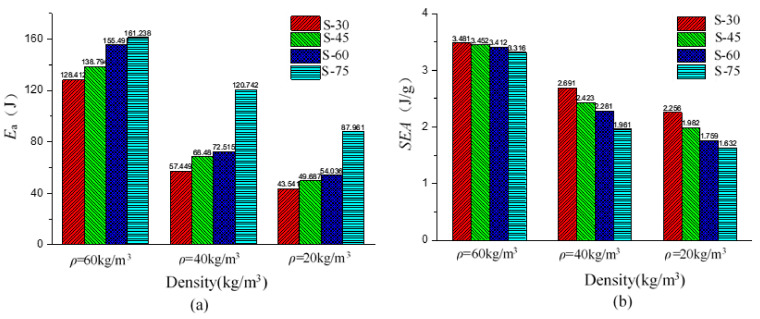
Effect of density and thickness on energy absorption characteristics (**a**) Absorbed energy *E*_a_ (**b**) Specific energy absorption *SEA*.

The higher EPP foam density not only led to higher mean crushing strength but also higher energy absorption. As shown in Figure 15a, when density increased from 20 kg/m^3^ to 60 kg/m^3^, the absorbed energy *E*_a_ of the EPP foam materials increased by 187.2% (54.06 J–155.491 J). When thickness increased from 30 mm to 75 mm, the absorbed energy *E*_a_ of the EPP foam materials increased by 25.5% (128.412 Mpa–161.238 Mpa).

Foam density also had a positive effect on specific energy absorption (*SEA*), whereas the foam thickness had a negative effect on *SEA*, as shown in Figure 15b. As EPP foam density increased from 20 kg/m^3^ to 60 kg/m^3^, *SEA* increased by 54.3% (2.256 J/g–3.481 J/g). When foam thickness increased from 30 mm to 75 mm, *SEA* decreased by 4.7% (3.481 J/g–3.316 J/g). A significant effect of foam density on SEA had also been observed in the study of the honeycomb panels filled with EPP foams [9], which was found in the honeycomb panels filled with PU foams. Compared to the study conducted by Mozafari and Molatefi [34], the SEA value of the PU foam with a density of 65 kg/m^3^ was 1.71 J/g, lower than that of the EPP foam with a density of 60 kg/m^3^ presented in this study.

**Figure 16:**
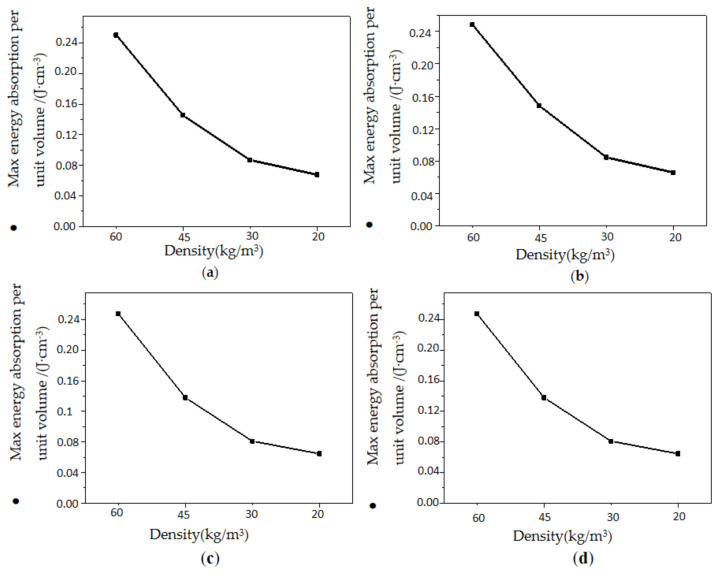
Maximum energy absorption per unit volume with different densities: (**a**) *t* = 30 mm; (**b**) *t* = 45 mm; (**c**) *t* = 60 mm; and (**d**) *t* = 75 mm.

**Figure 17:**
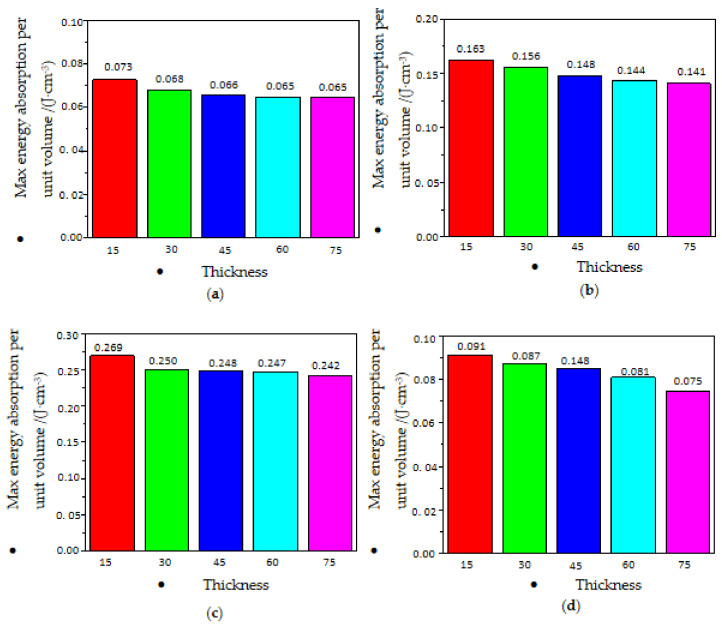
Maximum energy absorption per unit volume with different thicknesses. (**a**) *ρ* = 20 kg/m^3^; (**b**) *ρ* = 30 kg/m^3^; (**c**) *ρ* = 45 kg/m^3^; (**d**) *ρ* = 60 kg/m^3^.

EPP foams absorbed energy through the deformation of the material when subjected to compression load. The maximum absorbed energy per unit volume of polypropylene foams increased with an increase in EPP foam density but decreased with an increase in EPP foam thickness, as shown in Figure 16 and Figure 17, respectively. The denser the material, the larger the shape variable, and the more energy it would absorb per unit volume. Meanwhile, the loading speed selected for materials with different thicknesses was the same in the test, and a greater thickness indicated a smaller corresponding strain rate. Considering the sensitivity of EPP foam to the strain rate, the smaller the area under the stress—strain curve was, the lower the maximum energy absorption per unit volume became.

## 4. Conclusions

Herein, the crushing responses and mechanical characteristics of EPP foams with different densities and thicknesses under static compression were experimentally studied. The cushioning coefficient, mean crushing strength, energy absorption (*E*_a_), energy absorption efficiency (*E*_f_), SEA, and energy absorption per unit volume (*w*) were analyzed, and the following conclusions were drawn:

(1) The stress–strain curves of the EPP foams presented the same trend and were similar to the compression characteristics of other closed-cell foams. These curves can be divided into three stages: the linear elastic stage, the plateau stage, and the densification stage. The stress–strain constitutive relation of the EPP foam was a cubic function with a constant term.

(2) When thickness was constant, the mean crushing strength, energy absorption (*E*_a_), energy absorption efficiency (*E*_f_), *SEA*, and energy absorption per unit volume (*w*) increased, while the minimum cushioning coefficient decreased with increasing density. In the case of a constant density, a smaller thickness produced a larger *SEA*, a larger energy absorption efficiency (*E*_f_), and a smaller minimum cushioning coefficient. Within a certain range of strain rate, the influence of thickness on densification strain and the mean crushing strength was not obvious.

(3) By analyzing the microstructures observed by SEM of the samples before and after compression, the failure mechanism and instability process under the compression load were analyzed. The EPP foam exhibited considerable coverability after compression, potentially allowing further crushing energy absorption.

In this case, within a certain range of static compression conditions, there was an optimal thickness and density range for each cushioning material. In the optimization design of cushioning packaging, in order to prevent resource waste caused by excessive packaging or damage caused by insufficient packaging, the cushioning and energy absorption performance of foams with different thicknesses and densities should be fully compared, and an optimal scheme should be selected according to the comparison results. This will help to further understand the cushioning property and choose the right material while designing cushioning packages.

## Figures and Tables

**Table 1:** Geometric and crushing characteristics.

Specimen	Mass (g)	Peak Strength (MPa)	Mean Crushing Strength (MPa)	Densification Strain	Energy Absorption (J)	SEA (J/g)
S60-15	36.89	0.142	0.402	0.646	128.412	3.481
S60-30	40.21	0.144	0.397	0.631	138.794	3.452
S60-45	45.57	0.141	0.382	0.625	155.491	3.412
S60-60	48.62	0.118	0.268	0.619	161.238	3.316
S60-75	65.75	0.149	0.259	0.615	211.254	3.213
S45-15	23.95	0.065	0.259	0.664	70.681	2.951
S45-30	27.52	0.069	0.235	0.625	79.052	2.873
S45-45	35.74	0.087	0.226	0.616	94.078	2.632
S45-60	41.16	0.078	0.181	0.608	98.537	2.394
S45-75	63.87	0.069	0.160	0.602	150.158	2.351
S30-15	19.82	0.042	0.223	0.645	54.362	2.743
S30-30	21.35	0.044	0.144	0.623	57.449	2.691
S30-45	28.26	0.049	0.123	0.620	68.480	2.423
S30-60	31.79	0.043	0.101	0.614	72.515	2.281
S30-75	61.57	0.047	0.086	0.607	120.742	1.961
S20-15	18.32	0.031	0.201	0.658	42.521	2.321
S20-30	19.30	0.038	0.115	0.638	43.541	2.256
S20-45	25.07	0.034	0.104	0.632	49.687	1.982
S20-60	30.72	0.032	0.042	0.630	54.036	1.759
S20-75	53.89	0.029	0.027	0.626	87.961	1.632

## Data Availability

The data presented in this study are available on request from the corresponding author. The data are not publicly available due to privacy.
